# The Different Effects of Skeletal Muscle and Fat Mass on Height Increment in Children and Adolescents Aged 6–11 Years: A Cohort Study From China

**DOI:** 10.3389/fendo.2022.915490

**Published:** 2022-07-22

**Authors:** Dingting Wu, Liuhong Shi, Qiongying Xu, Yuanyuan Zeng, Xihua Lin, Xiaolin Li, Hanxin Zhao, Zhihong Zhu, Yeliu Fu, Hong Li, Xuehong Dong

**Affiliations:** ^1^ Department of Nutrition division, The Fourth Affiliated Hospital Zhejiang University School of Medicine, Yiwu, China; ^2^ Department of Ultrasound, The Second Affiliated Hospital of Zhejiang University School of Medical, Hangzhou, China; ^3^ Department of Medicine, Liangzhu Hospital, Hangzhou, China; ^4^ Department of Endocrinology and Metabolism, School of Medicine, Zhejiang University Affiliated Sir Run Shaw Hospital, Hangzhou, China; ^5^ Key Laboratory of Biotherapy of Zhejiang Province, Biomedical Research Center, Sir Run Run Shaw Hospital, Zhejiang University School of Medicine, Hangzhou, China; ^6^ Office of Health Monitoring and Statistics, Yiwu Center for Disease Control and Prevention, Yiwu, China

**Keywords:** skeletal muscle, fat mass, height increment, children, adolescent

## Abstract

**Objective:**

This study aimed to investigate the contribution of body composition including skeletal muscle mass (SMM) and body fat mass (BFM) to longitudinal growth among children and adolescents aged 6–11 years old.

**Methods:**

This cohort study was conducted from the annual health examination between 2019 and 2020. Annual height gain and weight gain and changes in SMM and BFM were calculated and compared between sexes, different nutritional status, and growth curve shifting mode. Spearman analyses and multiple linear regression analysis were performed to identify the impact of SMM, BFM, or body mass index (BMI) on height gain.

**Results:**

Of the 584 subjects, the annual height gains of boys (4.76 cm in the 6–9-year group and 4.63 cm in the 10–11-year group) were significantly lower than those of girls (5.48 and 5.74 cm, respectively). Spearman analysis showed that SMM gain and height gain were positively and significantly correlated in each examination of all children (*r* = 0.535 for boys and 0.734 for girls, *p* < 0.001). Conversely, BFM and height gains were negatively (*r* = -0.5240 for boys and -0.232 for girls, *p* < 0.001) correlated. Multiple linear regression analysis identified SMM gain as an independent predictor (95% CI: 1.20,1.44) of height gain after adjusting for age, gender, BMI, BFM gain, and percentage of body fat (PBF).

**Conclusion:**

SMM gains, rather than BFM gains, were associated with height gains in children and adolescents aged 6–11 years. Monitoring SMM changes in routine healthcare might motivate children and adolescents to achieve dietary and exercise recommendations, thereby growing taller without gaining excessive weight.

## Introduction

Longitudinal growth is an important indicator of health and nutrition in children’s routine healthcare. Several factors are involved in the regulation of children’s linear growth directly or indirectly, such as genes, nutritional status, hormonal level, and lifestyle. The definitions of nutritional status in Chinese students’ physical examination are generally based on body mass index (BMI), calculated from weight and height (1, 2). However, weight gain is composed of the gains in body fat mass (BFM) and fat-free mass (FFM), whose effects on linear growth are different or even opposite. Skeletal muscle mass (SMM) is the main component of FFM which is increasingly being considered as an important indicator of metabolic health. Previous studies (3–6) showed that SMM gains in children are positively associated with better bone mineral content, fewer metabolic risks, and improved neurodevelopment. However, research on SMM gain and linear growth’s relation is rare.

Short stature is the most common reason for referral to the pediatric endocrinologist. Height gain in children and adolescents is related to better nutrition, school competence, and less emotional disturbance (7–9), therefore drawing more and more attention in the clinical work of China. The latest report (10) showed that girls in South Korea, Vietnam, and some central Asian countries and boys in central and western Europe had the healthiest changes in anthropometric status over the past 3–5 decades because they had a much larger gain in height than they did in BMI.

Compared with substantial research on the cause and prevention of children’s obesity and overweight (11–14), there were much fewer studies focusing on height gain in children and adolescents. Earlier studies seemed to support the hypothesis that overnutrition not only produces a normal growth but also accelerates linear growth (15, 16). However, these data were based on inpatient or outpatient series, and the number of subjects was relatively small. Several cohort studies have demonstrated that growing up in a state of obesity demonstrated a smaller growth spurt during puberty, leading to an impaired final height (17–19). However, some of the series were taken from the obesity clinic, and most of the studies made the evaluation based on BMI measurement. Although in children and adolescents, BMI levels are strongly associated with FFM index as FFM makes up about 75% (girls) to 80% (boys) of the weight (20, 21), it should be noticed that among children with a BMI for age ≥85th percentile, BMI levels were strongly associated with the FM index (21). The role of BMI changes in defining height changes should be considered cautiously, and the effect of body composition on height gain can provide more precise information to improve healthier growth.

Therefore, the objective of this study is to investigate the association between BFM, FFM, and SMM changes and height gain from a cohort study between 2019 and 2020 in Yiwu City of China, sorting out the effect of body composition on longitudinal growth.

## Materials and Methods

### Subjects

The datasets in this cohort study were collected from the annual health examination records of 618 apparently healthy children aged 6–11 years old between 2019 and 2020 in Yiwu City of Zhejiang province, one of the economic developed regions in China. The study protocol was approved by the Human Ethics Committee of The Fourth Affiliated Hospital, Zhejiang University School of Medicine (China; approval no. K2020159). The students’ parents or guardians provided written informed consent for participating in the health examinations. Information on 34 students was not obtained because they were transferred to other schools and failed to attend the last investigation. Ten students were also excluded because of invalid data.

### Measurement and Procedures

Two well-trained nutritionists from the Fourth Affiliated Hospital of Zhejiang University Medicine School and five physicians from a community hospital conducted health examinations of all students. Physical exam items including eyes, teeth, ears-nose-throat, head-neck, heart, chest-lung, and abdomen according to the Chinese Standards for physical examinations (22) were recorded on student health examination records. Anthropometrics including height, weight, and visual acuity were measured by community doctor prior to the physical examination dates. Body weight was measured to the nearest 0.1 kg through body composition examination and height to the nearest 0.5 cm using a stadiometer (Seca 704, Hangzhou, China) in school settings. The first and second evaluations were both carried out in late September while students were dressed in minimal clothing.

Body composition was measured by InBody 770 (InBody Co., Ltd., Seoul, Korea). InBody 770 is a model of bioelectrical impedance analysis (BIA) with an eight-point tactile electrode system. Students were asked to take off their shoes and socks. After wiping down their hands and feet, students stepped onto the machine and stood upright, positioning their bare feet on the foot electrodes. Weight was automatically measured. Then students grabbed the handles and placed their thumbs on the oval electrodes. A small electrical current was passed through the body, while the resistance of the body’s five cylinders (left arm, right arm, torso, left leg, and right leg) was measured, and the in-built equation was used to convert the input impedance to body composition estimates including SMM (kg), FM (kg), and so on.

Students with complete follow-up data were categorized into underweight, normal weight, overweight, and obese groups according to the nutritional status by BMI based on the Chinese national standard of screening for overweight and obesity among school-age children and adolescents (1, 2).

The subjects’ length/height for age and gender were calculated according to the growth curves published by the World Health Organization (WHO) (23) and were expressed in z-scores (-3, -2, -1, 0, + 1, +2, +3). The growth curve’s shift for each subject in Child Growth Chart from 2019 to 2020 were monitored.

### Statistical Analysis

The computer software statistical package programmed SPSS version 20 was used to analyze the data (IBM Corporation, New York, NY, USA). Univariate analyses were conducted by independent sample t-test or non-parametric analysis or chi-square (χ^2^) test or bivariate linear correlation. Continuous data were expressed as means ± standard derivations whereas categorical variables were expressed as frequency and percentage. Independent sample t-test was applied for the data which was normally distributed while non-parametric analysis was used for non-normal distribution. Spearman’s analyses were preformed to estimate the correlations of height gain with weight gain, SMM gain, BFM gain, or other clinical continuous variables. The χ^2^ test was used for between-group comparisons of categorical variables. The variables presenting significant relationships (*p* < 0.05) with height gain were then included into multiple linear regression analysis to assess the association of these factors with height gain.

## Results

### Descriptive Data

In total, 618 typically developing students enrolled in the school year 2019 at baseline, and the remaining 584 (94.5%) of their follow-up datasets in 2020 were collected. Three hundred fifteen boys (53.9%) and 269 girls (46.1%) who had complete data were further stratified according to the different age categories as follows (24): the prepubertal stage 6–9-year group and the early pubertal stage 10–11-year group. The descriptive data including height, weight, annual height gain, annual weight gain, BFM, BMI, FFM, SMM, and annual gains of SMM and BFM are shown in **Table 1**. In general, the annual height gains of boys (4.76 cm in the 6–9-year group and 4.63 cm in the 10–11-year group) were significantly lower than those of girls (5.48 and 5.74 cm, respectively) in this cohort. Girls aged 10–11 years had the highest annual height gain of 5.74 cm, while boys aged 10–11 years had the highest annual weight gain of 7.43 kg in average. The annual gains of BFM and BMI among boys were higher than those of girls in both age categories. However, the average annual gains of SMM (1.69 kg) and FFM (2.91 kg, *p* < 0.05) among 6–9-year-old girls were higher than those of boys (1.55 and 2.64 kg, respectively) in the same age category, consistent with the different annual height gain between the two sexes.

**Table 1:** Anthropometric and body composition characteristics of participants by sex and age. Values = mean ± SD.

Variable	Boys				Girls			
	6-9y		10-11y		6-9y		10-11y	
N	243	243	72	72	212	212	57	57
Year	2019	2020	2019	2020	2019	2020	2019	2020
Age (years)	8.04±1.23	9.14±1.19	10.49±0.53*	11.65±0.51*	8.28±1.16	9.34±1.15	10.53±0.50*	11.68±0.47*
Height (cm)	130.64±8.55	135.37±8.15	143.94±6.50*	148.58±7.39*	130.24±8.35	135.75±8.62	145.77±6.84*	151.49±6.66*
Annual Height Gain (cm)		4.76±1.91		4.63±2.23		5.48±2.14#		5.74±2.52*#
Weight (kg)	27.83±6.80	32.81±8.85	37.07±9.67*	44.50±12.71*	26.05±6.55	30.67±8.38	34.63±7.01*	41.16±7.98*
Annual Weight Gain (kg)		5.02±2.73		7.43±3.94*		4.62±2.31		6.56±2.26*
BFM (kg)	5.33±3.68	7.70±5.26	9.17±6.05*	11.94±7.56*	4.70±3.46	6.39±4.37	7.06±4.04*	9.45±4.56*
AG-BFM (kg)		2.37±2.14		2.77±2.66		1.69±1.54		2.39±1.76*
SMM (kg)	11.17±2.27	12.72±2.59	14.49±2.64*	17.07±3.62*	10.51±2.25	12.19±2.79	14.11±2.43*	16.47±2.47*
AG-SMM (kg)		1.55±0.77		2.68±1.56*		1.69±0.97		2.37±0.98*
FFM (kg)	22.45±3.81	25.10±4.31	27.96±4.32*	32.50±6.18*	21.39±3.77	24.25±4.72	27.54±4.02*	31.72±4.22*
AG-FFM (kg)		2.64±1.15		4.58±2.55*		2.91±1.55#		4.15±1.71*
BMI (kg/m^2^)	16.09±2.59	17.61±3.34	17.65±3.71*	19.94±4.47*	15.19±2.42	16.41±2.80	16.25±2.57*	17.81±2.75*
PBF (%)	17.70±7.81	21.41±9.15	22.58±9.37*	24.66±9.47*	16.52±7.91	19.19±7.81	19.26±7.22*	21.98±6.52*

BFM, Body Fat Mass; AG-BFM, Annual gain of BFM; SMM, Skeletal Muscle Mass; AG-SMM, Annual gain of SMM; FFM, Fat Free Mass; AG-FFM, Annual gain of FFM; BMI, Body Mass Index; PBF, Percentage of Body Fat. * Comparison between the 6-9 years and the 10-11 years age category (p < 0.05). # Comparison between boys and girls in the same age category (p < 0.05).

**Figure 1:**
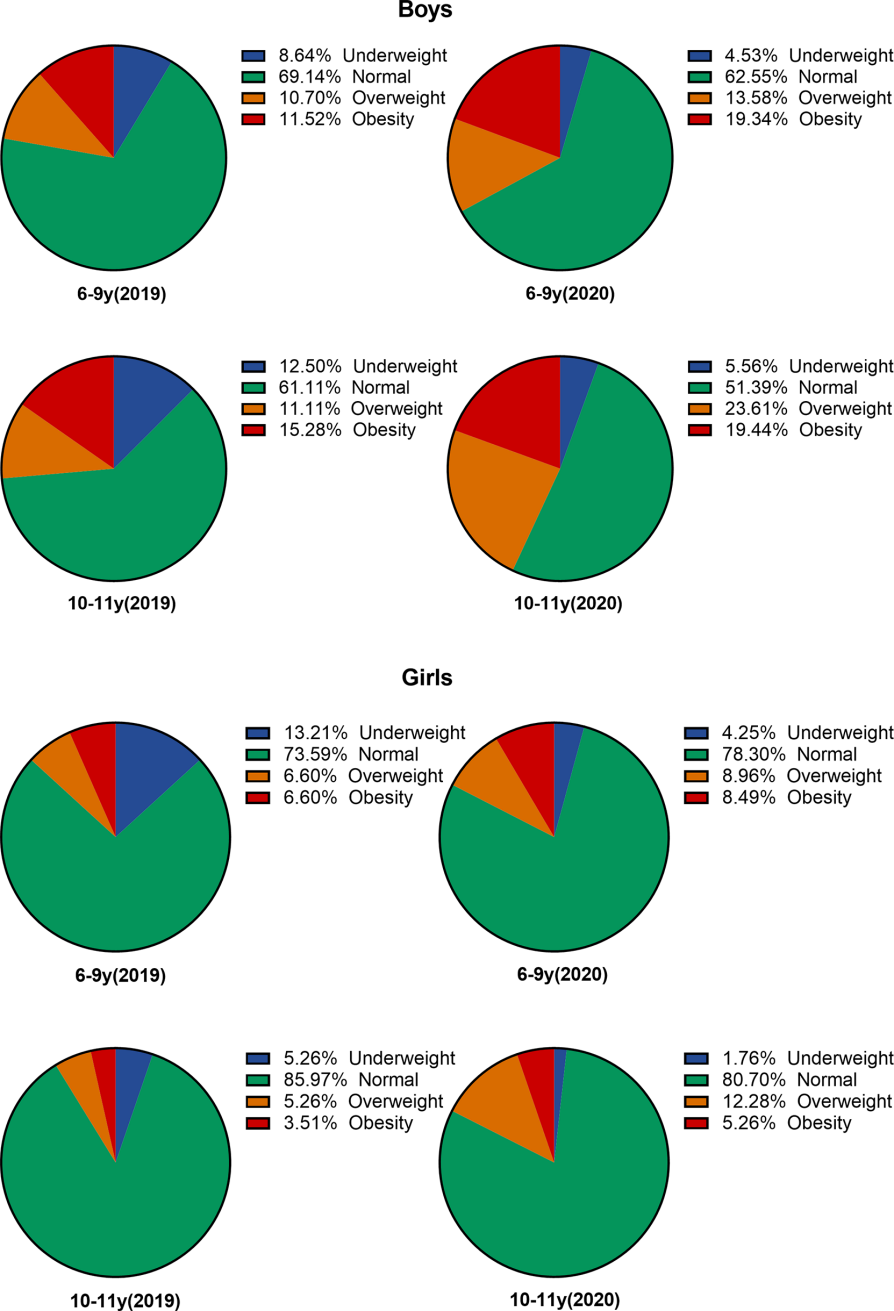
The prevalence of different nutritional status for both sexes in 2019 and 2020.

The prevalence of underweight, normal weight, overweight, and obesity according to the nutritional status by BMI in two age categories among both sexes is illustrated in **Figure 1**. During the 2-year follow-up, 10-11-year-old boys in overweight and obese group increased by 12.5% and 4.2%, respectively, while girls in the same age category had 7.0% and 1.8% increment. For 6–9-year-old boys, the prevalence of obesity increased from 11.5% to 19.3%, while that of girls increased only from 6.6% to 8.5%.

### Correlation of Height Gain and Body Composition

**Figure 2:**
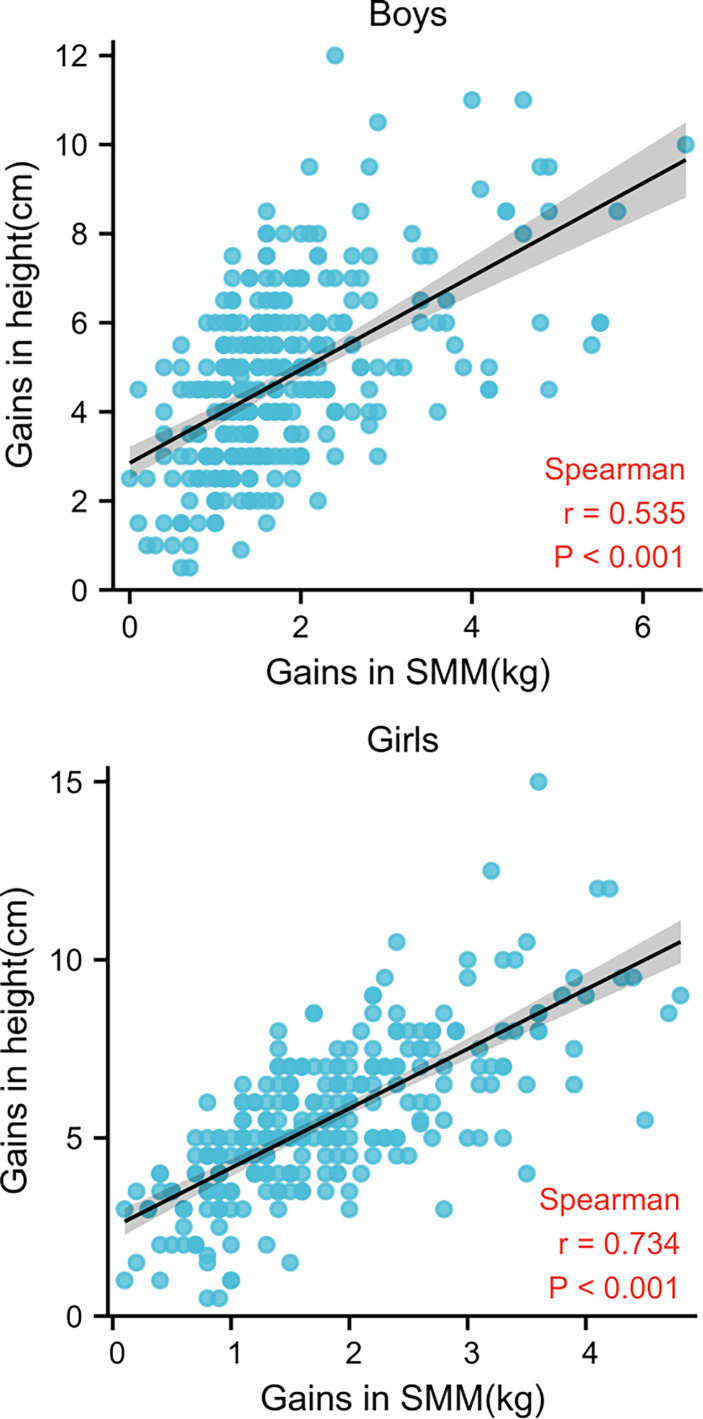
Spearman’s correlation between gains in height and SMM in both sexes.

**Figure 3:**
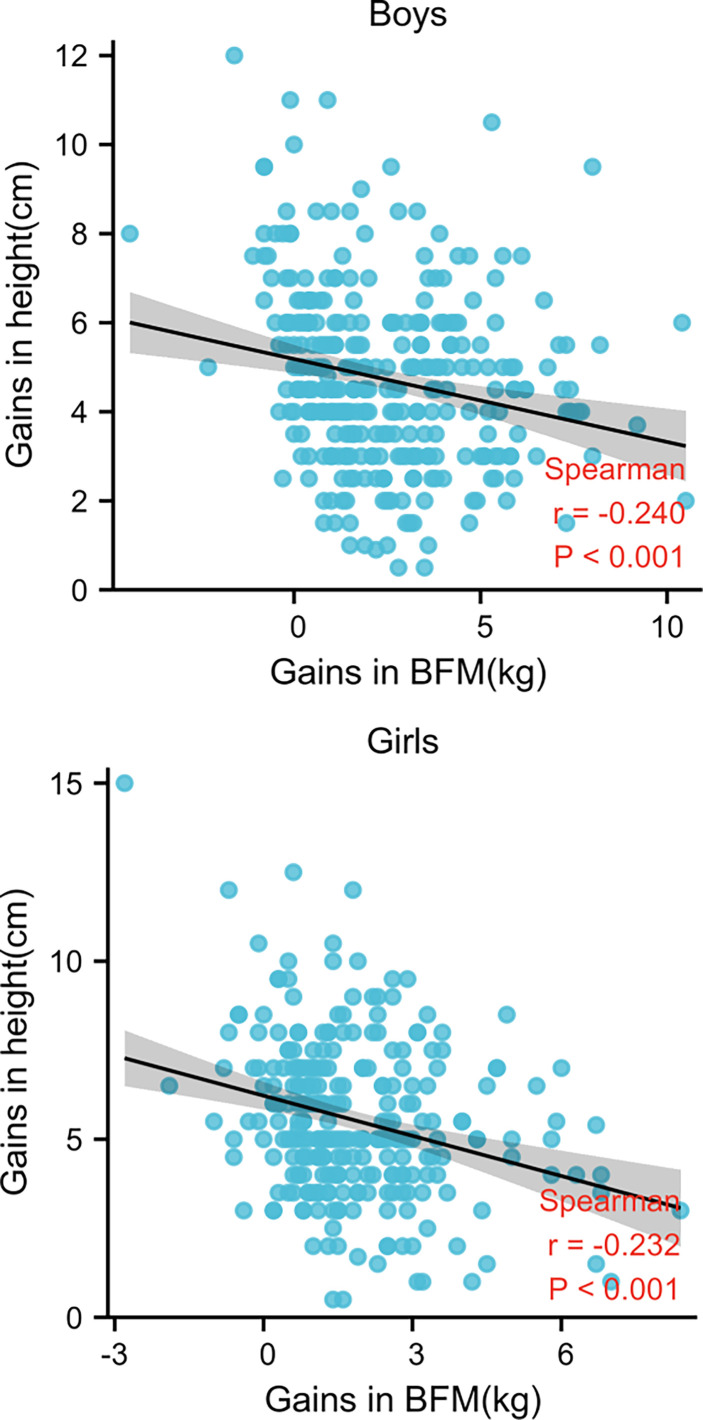
Spearman’s correlation between gains in height and BFM in both sexes.

**Figure 2** demonstrates that height gain and SMM gain were positively and significantly correlated in each examination of all children (*r* = 0.535 for boys and 0.734 for girls, *p* < 0.001). Conversely, height and BFM gains were negatively (*r* = -0.5240 for boys and -0.232 for girls, *p* < 0.001) correlated (**Figure 3**).

Multiple linear regression analysis was then applied, and identified SMM and BFM gains were linearly correlated with height gain (**Table 2**). This analysis also demonstrated SMM gain as an independent predictor (95% CI: 1.20,1.44) of height gain after adjusting for age, gender, BMI, BFM gain, and PBF (**Table 2**).

**Table 2:** The linear regression analysis of height gain and its correlative factors.

Covariates	Univariate analysis			Multivariate analysis		
	exp (B)	95% CI	*p* value	exp (B)	95% CI	*p* value
Age, years	-0.07	-0.19, 0.05	0.23			
Gender, Female	0.81	0.47, 1.15	<0.001	0.54	0.29, 0.79	<0.001
BMI, kg/m^2^	0.02	-0.03, 0.07	0.438			
Gains in SMM, kg	1.19	1.06, 1.32	<0.001	1.32	1.20, 1.44	<0.001
Gains in BFM, kg	-0.27	-0.35, -0.19	<0.001	-0.38	-0.44, -0.31	<0.001
PBF, %	-0.01	-0.03, 0.01	0.32			

BMI, Body Mass Index; SMM, Skeletal Muscle Mass; BFM, Body Fat Mass; PBF, Percentage of Body Fat.

### Correlation Between Height Increment and SMM Changes in Different Parts of the Body

**Figure 4:**
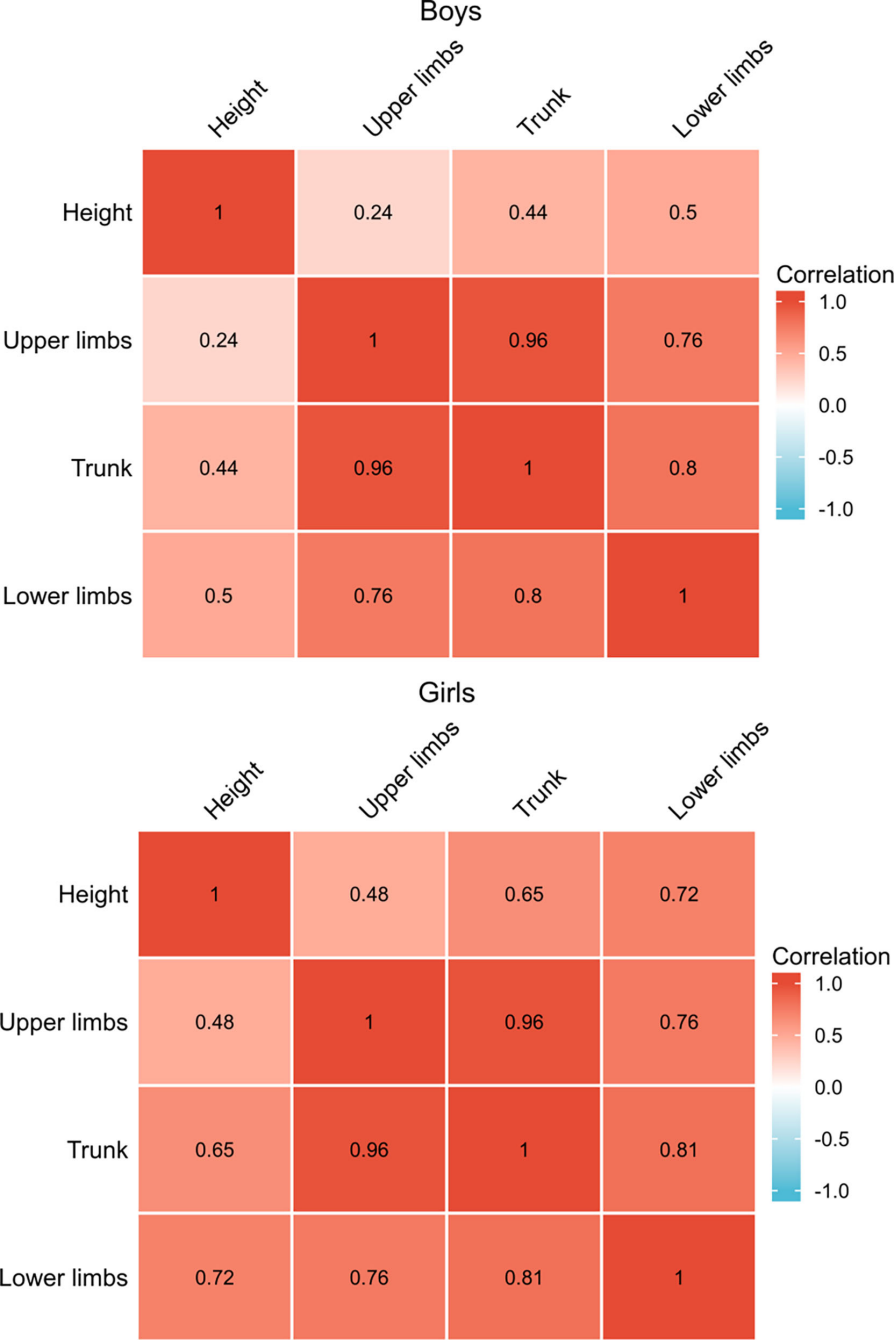
Spearman’s correlation analysis with height gain and gains of SMM in different parts of the body.

To decipher the influence of SMM changes in different parts of the body, we analyzed the correlation of upper limbs, lower limbs, and trunk SMM gains specifically with height gain by one-way ANOVA (**Figure 4**). The significant statistical results (correlation coefficient, P-value) were as follows: SMM gains of lower limbs in boys (*r* = 0.5, *p* < 0.05) and girls (*r* = 0.72, *p* < 0.05), trunk SMM gain in girls (*r* = 0.65, *p* < 0.05). The correlation coefficients in the upper limbs and trunk SMM increment of boys and upper limbs of girls with height gain indicate a weak positive linear relationship (*r* < 0.5).

### Correlation Between Changes of SMM, BFM, and Height in Different Nutritional Status

**Figure 5:**
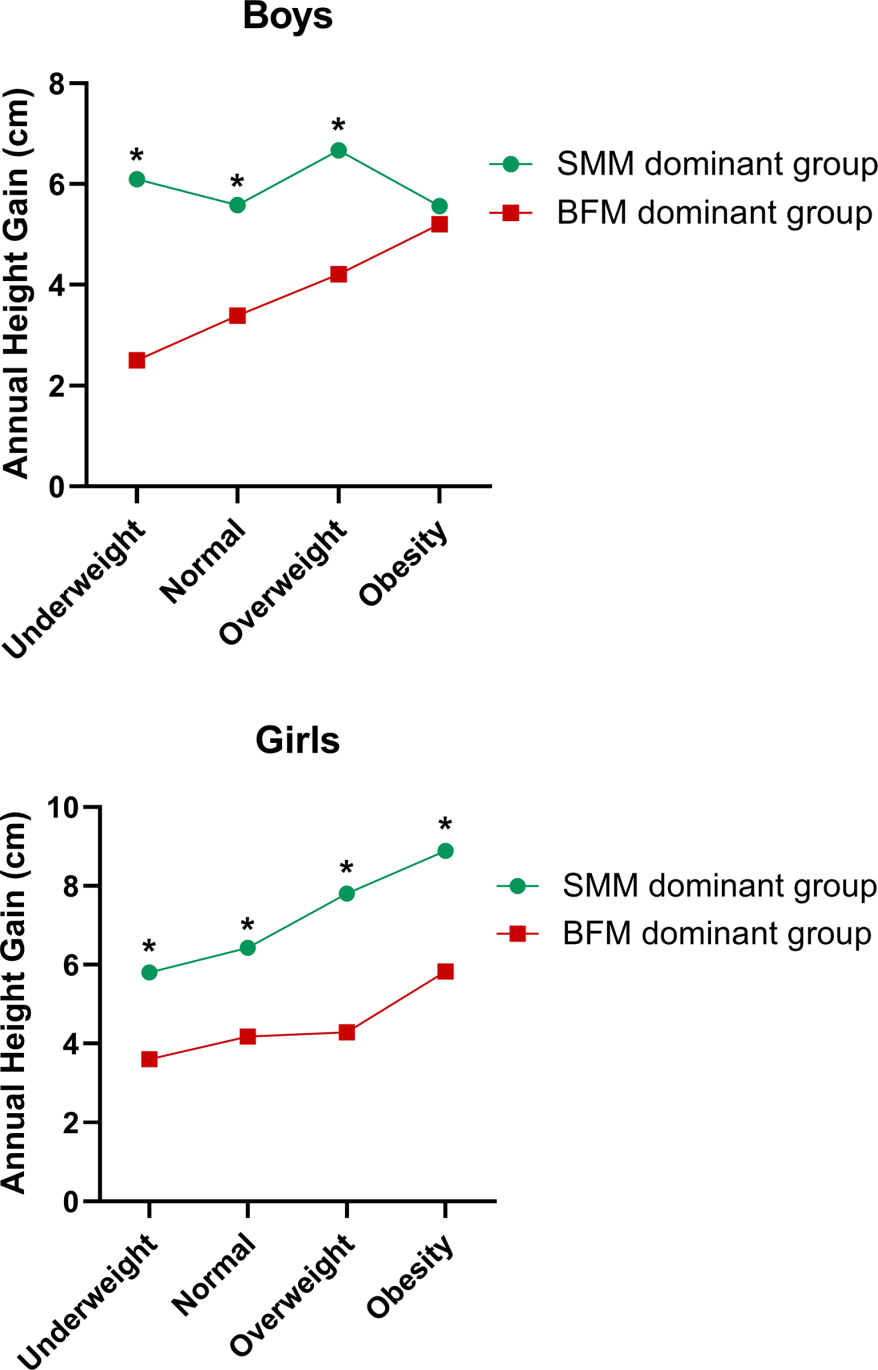
Correlation between changes of SMM, BFM, and annual height gain in different nutritional statuses. *Significant difference to the BFM dominant group (*p* < 0.05).

To observe the changes in SMM, BFM, and height gain further in different nutritional statuses, we divided the subjects into another two groups as follows (**Figure 5**): the SMM gain dominant over BFM gain group (46.2%) defined as SMM gain (kg) minus BFM gain (kg) > 0, and the BFM dominant over SMM group (53.8%) defined as SMM gain (kg) minus BFM gain (kg) <0. Girls accounted for 52.6% of the SMM gain dominant group (*p* < 0.05 vs. boys), while the percentage of boys was 58.6% in the BFM dominant group (*p* < 0.05 vs. girls). For girls in each different nutritional status, the height gain of the SMM gain dominant group was always superior to that of the BFM gain dominant group (*p* < 0.05). For boys with obese status, there was no difference in height gain between two groups. However, in the normal, overweight, and even underweight status of boys, the SMM gain dominant group had significantly higher height gain than the BFM dominant group (*p* < 0.05).

### Effect of SMM and BFM Gains on Growth Curve’s Shift

**Figure 6:**
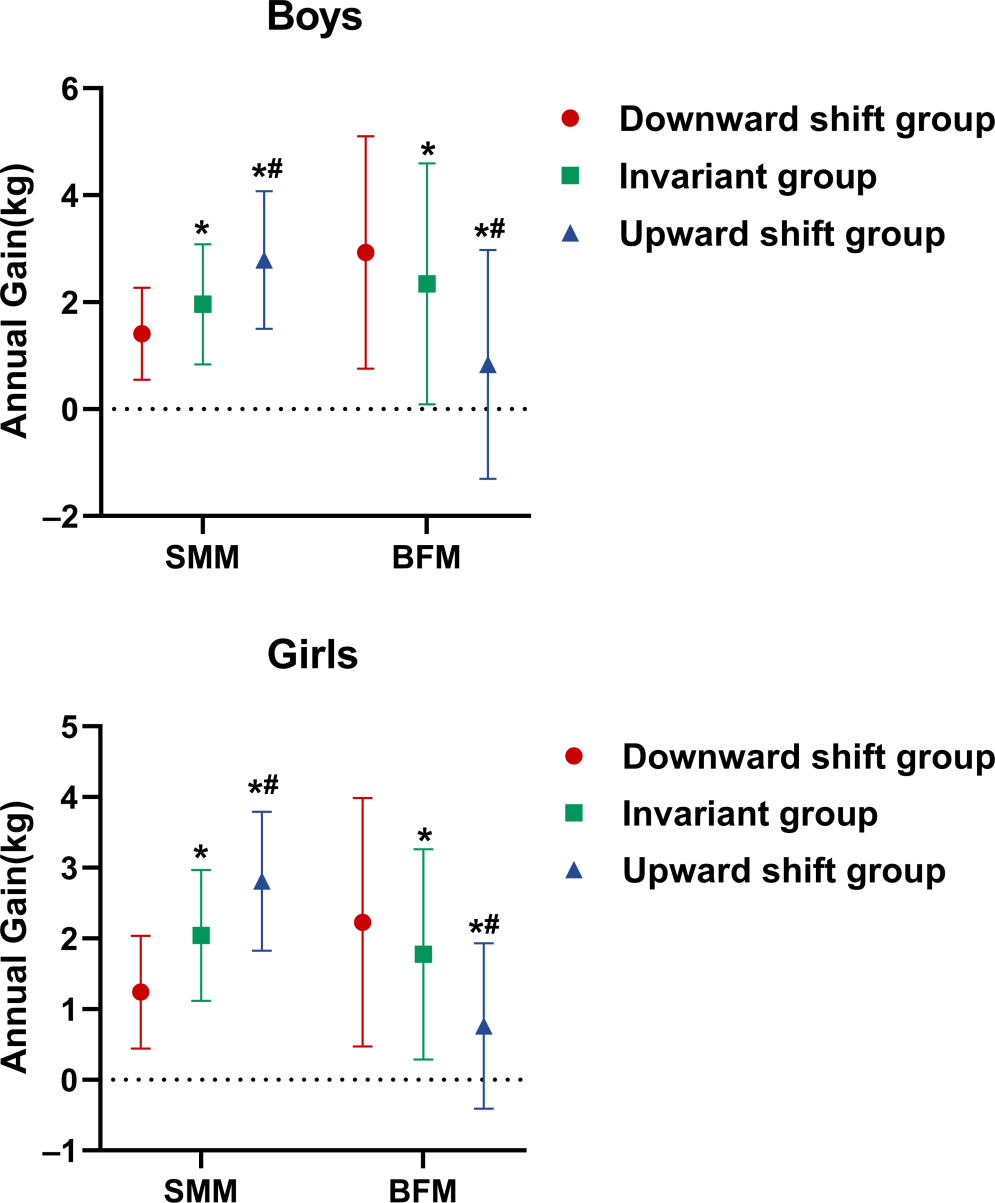
Correlation of annual SMM and BFM gains with growth curve’s shifting mode in the 2-year follow-up. *Significant difference to the downward shift group (*p* < 0.05). ^#^Significant difference to the invariant group (*p* < 0.05).

Using the anthropomorphic measures obtained in the 2-year follow up, the subjects’ length and height for age and gender were calculated according to the growth curves published by the World Health Organization (WHO) (23) and were expressed in z-scores(-3, -2, -1, 0, + 1, +2, +3). We monitored the growth curve’s shift for each subject in the Child Growth Chart from 2019 to 2020 and then divided them into three groups (**Figure 6**): the downward shift group (the z-score slid by at least one interval), the invariant group (the z-score had no change), and the upward shift group (the z-score climbed by at least one interval). Among these three groups, the annual SMM gain increased gradually from 1.41, 1.96, to 2.79 kg in boys and 1.24, 2.04, to 2.81 kg in girls. By contrast, the BFM gain reduced gradually from 2.93, 2.34, to 0.84 kg in boys and 2.23, 1.78, to 0.76 kg in girls. Compared with the invariant and downward shift groups, the annual SMM gain in the upward shift group was significantly higher for both sexes. On the contrary, the annual BMF gain in the downward shift group was significantly higher than those in the other two groups.

## Discussion

This cohort study was conducted in Yiwu, one of the economic developed cities located in south-eastern China with relatively faster height and weight growth in children and adolescents (25). As medical practitioners’ and caregivers’ interest in the nutritional status of children has increased, research on height gain, besides obesity or weight gain, has become active. Our main finding revealed that SMM gains, rather than BFM gains, were associated with height gains in children and adolescents aged 6–11 years. To our knowledge, this is the first study to sorting out the effect of body composition on longitudinal growth.

Previous studies with a relatively small number of subjects showed that overnutrition not only produces a normal growth but also accelerates linear growth (15, 16) based on inpatient or outpatient series. A study taken from an obesity clinic explored the association between obesity and height throughout childhood and adolescence (17); the obese subjects were taller than average in childhood, but the growth advantage gradually decreased and the final adult height of obese and normal subjects was equal. A recent Taiwan cohort study (19) also illustrated that obese subjects between 9 and 13 years of age tended to have higher linear growth velocity than subjects with normal BMI. However, the Swedish population–based longitudinal growth study (18) concluded that overnutrition between 2 and 8 years of age will not be beneficial from a final height point of view, as the temporary increase in height gain will be compensated by an earlier pubertal maturity and a subnormal height gain in adolescence.

Most of these studies were based on BMI, without a concomitant consideration of body composition’s effect (including SMM, FFM, and BFM) on linear growth. Although BMI is widely used as a surrogate measure of adiposity, it is a measure of excess weight relative to height, rather than excess body fat. BMI levels among adults are highly correlated with %body fat (26, 27), but the associations among children and adolescents have been more variable (21, 28) and relatively weak correlations have been reported in several subgroups. These weaker associations among children and adolescents may be attributable to the asynchronous changes that occur in the levels of FM and FFM during growth (21). On the other hand, BIA is a cost-effective and convenient method to evaluate body composition, with a 2-min testing time and good consistency with dual x-ray absorptiometry (DXA), magnetic resonance imaging, computed tomography, and ultrasound results but with no radiation exposure (29–31). We made the measurements on late September in the 2-year follow-up to avoid the influence factors such as temperature and humidity. The average BMI and FFM of 6–11-year-old boys and girls in our data were consistent with those of a large-scale population-based multicenter data from the China Child and Adolescent Cardiovascular Health (CCACH) Study using DXA to evaluate the body composition (32). Our FM and SMM data of both boys and girls were lower in average than those of the CCACH data probably due to the relatively fewer southern students included in the CCACH study and the possible different diet habits and thus muscle mass between students from north to south.

Our results demonstrated that SMM gain and height gain were positively and significantly correlated in each examination of these 6–11-year-old students, while BFM and height gains were negatively correlated. Furthermore, we identified SMM gain as an independent predictor of height increment after adjusting for age, gender, BMI, BFM gain, and PBF. To be more specific, SMM gains of lower limbs in boys and girls and trunk SMM gains in girls were most significantly correlated with height gain. Skeletal muscle is a tissue capable of modifying its structure and metabolic properties (33), whose development was affected by several postnatal environmental factors such as dietary protein (34), physical activity (35), chronic diseases (36), and obesity. It is well documented that skeletal muscle has many physiological functions through biomechanical stress and multiple myokines (37). Maintaining optimal skeletal muscle mass in childhood may improve the peak muscle mass and bone strength and exert beneficial effects on cardiovascular health in adulthood (3, 38). However, no study has explored the relation of SMM and height gains yet. Our data emphasized that SMM gains, especially the lower limb SMM gains for both boys and girls, were important to achieve greater height increment. Data from the Physical Fitness and Health Research of Chinese school students from 1985 to 2010 (39) showed that the trends in upper and lower limbs SMM were consistent with those of grip strength and distance of standing long jump. Moreover, performance of moderate-to-vigorous physical activity for ≥5 days per week for ≥60 min per day was associated with the SMM index score of Japanese children (40). These findings together with ours indicated that physical activity plays a vital role in the development of skeletal muscle in youth, and moderate-to-vigorous physical activity is important for the development of SMM and hence height gain.

Growth hormone, insulin-like growth factor 1, insulin, ghrelin, and sex steroids act synergistically to stimulate muscle protein synthesis and reduce its oxidation rate while leading to a positive protein balance and, consequently, muscle accretion (41) during childhood and adolescence. A recent study including 3991 children aged 8 years found an association between higher protein intake and higher FFM measured by DXA (42). On the other hand, one study revealed that a higher protein intake was associated with increased FM (43) and risk of obesity in early childhood (44). Protein intake above the amount needed for growth appeared to stimulate adipogenesis and inhibit lipolysis in children (45). Research has shown that obesity contributes to low muscle mass and weakness (46); children and adolescents with obesity exhibit low relative strength to body mass (47), impaired muscular fitness (48), and reduced neuromuscular activation capacity (49) when compared to their non-obese counterparts. In our study, there was no deference in height gain for boys in obese status between the SMM dominant and BFM dominant groups. Furthermore, among the growth curve downward shift group, the invariant group, and the upward shift group, the annual SMM gain increased gradually in boys and girls whereas the BFM gain reduced in a step-wise manner. Taken together, personalized approaches including dietary and exercise recommendations should be considered to balance the SMM and BFM gains, thus achieving the maximum benefits for growth.

The Taiwan cohort study (19) also showed that 9–13-year-old underweight girls had higher linear growth, indicating that puberty may dominate over BMI as the main contributor to high growth velocity in girls with underweight BMI emerging into pubertal age. Our cohort data showed that for 6–11-year-old underweight girls, the height gain of the SMM gain dominant group was still superior to that of the BFM gain dominant group, highlighting that SMM gain could improve height gain, even in a malnutritional state.

### Limitations

This study has some limitations that need to be addressed. First, other factors which might affect height gains such as exercise, diet, and underlying diseases were not collected in our datasets. Second, the timing of puberty onset is essential to evaluate height gain, but no physical measurements of puberty were recorded in the present study. A large population-based study showed that the overall detection rates of precocious puberty of boys and girls were 9.53% and 23.07%, respectively, and earlier pubertal development was positively associated with obesity and central obesity in Chinese children (50). Since girls are known to mature earlier than boys, it is likely that puberty contributed to the outcome that the annual height gains of boys were significantly lower than those of girls. However, we analyzed our data in different nutritional status and growth curve shifting mode, and tested the SMM, BFM, and height changes dynamically, resulting in the similar conclusion of the close relation between SMM gains and height increment. Third, we did not follow the subjects for a longer period. Future studies should be conducted to evaluate the associations between body composition and growth velocity with more information such as exercise time, diet habits, and time of puberty until the subjects reach their final heights.

## Conclusions

This cohort study revealed that SMM gains, rather than BFM gains, were associated with height gains in children and adolescents aged 6–11 years. SMM gain was identified as an independent predictor of height increment. Our study has strengths in deciphering the body composition’s effects on height gain dynamically in different nutritional statuses. Monitoring SMM changes by non-invasive BIA in routine healthcare might be a key method to motivate children and adolescents to achieve the personalized approaches including dietary and exercise recommendations, thus growing taller without gaining excessive weight. It is time to make long-term strategic plans for the government to respond to the rising rates of overweight, obesity, and sarcopenia in our children and adolescents and, hence, to improve healthier growth.

## Data Availability Statement

The raw data supporting the conclusions of this article will be made available by the authors, without undue reservation.

## Ethics Statement

The studies involving human participants were reviewed and approved by the Ethics Committee of The Fourth Affiliated Hospital Zhejiang University School of Medicine. Written informed consent to participate in this study was provided by the participants’ legal guardian/next of kin.

## Author Contributions

DW and LS designed the research. DW, QX, and YZ collected the subjects’ data. XLL, ZZ, and YF contributed to the technical assistance. DW, LS, and ZZ analyzed the subjects’ data. LH and XD were major contributors to research and academic guidance. DW, XD, LH, XHL, and HZ interpreted the subjects’ data. All authors contributed to the article and approved the submitted version.

## Funding

This work was supported by grants from the Foundation of Zhejiang Provincial Education Department (Grant No. Y201942039), Science and Technology program of Yiwu Science and Technology Bureau (Grant No. 20-3-115), Zhejiang Provincial Natural Science Foundation of China (Grant Nos. LY21H070002 and LQ21H070002), and Medicine and Health Science Technology Project of Zhejiang Province (Grant No. 2021RC081).

## Conflict of Interest

The authors declare that the research was conducted in the absence of any commercial or financial relationships that could be construed as a potential conflict of interest.

## Publisher’s Note

All claims expressed in this article are solely those of the authors and do not necessarily represent those of their affiliated organizations, or those of the publisher, the editors and the reviewers. Any product that may be evaluated in this article, or claim that may be made by its manufacturer, is not guaranteed or endorsed by the publisher.
